# The importance of the eyes: communication skills in infants of blind parents

**DOI:** 10.1098/rspb.2013.0436

**Published:** 2013-06-07

**Authors:** Atsushi Senju, Leslie Tucker, Greg Pasco, Kristelle Hudry, Mayada Elsabbagh, Tony Charman, Mark H. Johnson

**Affiliations:** 1 Centre for Brain and Cognitive Development, Birkbeck, University of London, Malet Street, London WC1E 7HX, UK; 2 Department of Psychology, King's College London, Institute of Psychiatry, London, UK; 3 Olga Tennison Autism Research Centre, School of Psychological Science, La Trobe University, Australia; 4 Faculty of Medicine, Department of Psychiatry, McGill University, Canada

**Keywords:** infant development, social communication, social cognition, visual impairment, cognitive development, parent–child interaction

## Abstract

The effects of selectively different experience of eye contact and gaze behaviour on the early development of five sighted infants of blind parents were investigated. Infants were assessed longitudinally at 6–10, 12–15 and 24–47 months. Face scanning and gaze following were assessed using eye tracking. In addition, established measures of autistic-like behaviours and standardized tests of cognitive, motor and linguistic development, as well as observations of naturalistic parent–child interaction were collected. These data were compared with those obtained from a larger group of sighted infants of sighted parents. Infants with blind parents did not show an overall decrease in eye contact or gaze following when they observed sighted adults on video or in live interactions, nor did they show any autistic-like behaviours. However, they directed their own eye gaze somewhat less frequently towards their blind mothers and also showed improved performance in visual memory and attention at younger ages. Being reared with significantly reduced experience of eye contact and gaze behaviour does not preclude sighted infants from developing typical gaze processing and other social-communication skills. Indeed, the need to switch between different types of communication strategy may actually enhance other skills during development.

## 1. Introduction

Human infants are highly sensitive to adults’ communicative signals, such as use of eye contact, from the first few days after birth [1]. Within the first year, eye contact modulates infants’ learning about novel faces [2], gaze-following ability [3] and degree of cortical activation during face perception [4]. Atypical eye-contact behaviour is among the most characteristic early symptoms in autism spectrum disorders (ASDs) [5], which involve profound impairment in the development of social-communication skills.

A fundamental question about functional brain development is the effect of the postnatal environment. For example, what is the role of face-to-face communication in the development of gaze processing, the development of social skills and the development of the brain in general? One approach to this question is to study naturally occurring variability in postnatal social environments. For example, profound institutional deprivation [6] or congenital blindness [7] in early infancy reportedly affects social skills development and increases the prevalence of autistic-like behaviours. However, cases such as these involve a pervasive lack of social or visual input, making it difficult to more specifically understand which components of experience are critical for the effects observed.

By contrast, the sighted infants of blind parents (SIBPs) experience a more specifically different mode of social communication with their carers. In particular, because blind carers cannot perceive infants’ direction of gaze, SIBP are likely to have substantially reduced experience of eye contact and gaze-related behaviour from interaction with the parent than other infants. This contrasts with institutional deprivation, which involves a reduction in any form of social interaction, and congenital blindness, which involves reducing all visual experience. Studying the early development of SIBP provides an opportunity to examine the effects of selectively different early experience of eye contact and gaze behaviour on the development of social communication. To date, only two behavioural observation studies have been conducted with SIBP [8,9], both of which reported overall typical development in the cases presented. Both observations also reported that parents flexibly use touching, sounds and vocal communication to maintain a typical level of parent–child interaction (PCI). No studies have examined cases of SIBP with quantitative experimental or standardized measurements. The current study therefore presents the first empirical, systematic and longitudinal investigation of the development of social-communication skills in SIBP. We have assessed five SIBPs from early infancy through to toddlerhood, using experimental tasks, systematic observation of their behavioural development and systematic analyses of PCI.

Three viewpoints on the postnatal functional development of eye gaze processing have been described [10]. The nativist perspective, for example, proposes the existence of an innate module called the eye direction detector, which is to a large extent independent of postnatal experience [11]. This perspective predicts no effect of parental sight and the use of their gaze in social communication on the development of infant gaze-processing skills. By contrast, interactive specialization [10,12] assumes that infants are born with widespread connections between cortical and subcortical regions [12], and that input from subcortical routes interacts with architectural biases in the brain to form specialized networks for gaze processing. This theory predicts that SIBP could develop different forms of specialization, depending on unique input from their blind parent. Finally, the affective learning viewpoint [13–15] further emphasizes the role of postnatal experience, especially in the role of the reward value of eye contact which could emerge as a result of extensive exposure to the co-occurrence of eye contact and a wide variety of positive experiences through social interaction and communication [16], or the effect of social reinforcement on the development of infants’ gaze-following behaviour [14,15]. From this position, SIBP could fail to develop the usual type of expertise and interest in adults’ gaze because their use of eye contact and gaze processing are not reciprocated by blind parents and therefore do not become rewarding.

## 2. Material and methods

Participants were five sighted infants (two male) of blind mothers, including a pair of siblings (SIBP3 and 5) and 51 infants (21 male) of sighted parents. SIBP were recruited through charities, online communities of parents and personal contacts. In all five cases, the blind mothers were the infant's primary carers. While the degree and the cause of visual impairment in the blind mothers varied, all had experienced profound visual impairment for at least 15 years at the time of testing and their extent of visual impairment severely affected face-to-face communication with their infants (see the electronic supplementary material). Parent–infant dyads visited our centre twice, once between six and 10 months and then again between 12 and 15 months. These age points were selected to coincide with the availability of control data from infants of sighted parents. We then visited the dyads at home when the infants were aged between 24 and 47 months, to follow up their longer-term outcome of social, communicative and cognitive development [17–19]. One of the infants also had a partially sighted father, whereas the other four had a sighted father. Details of the familial environments of these infants are provided in the electronic supplementary material. The comparison infants with sighted parents were recruited from a volunteer database at the Centre for Brain and Cognitive Development, Birkbeck, as part of the *British Autism Study of Infant Siblings* (*BASIS:* www.basisnetwork.org.uk). These infants also attended two laboratory visits and completed the same tests as the SIBP group.

At each visit, infants completed two eye-tracking experiments of gaze-processing and behavioural assessments of social-communicative and cognitive development, and the dyads were recorded during naturalistic PCI. Data from these tasks were then compared with those from the same assessments conducted with a large group of sighted infants of sighted parents. SIBP infants were then followed up at a home visit between 2 and 4 years of age to assess their longer-term development.

In the two experimental tasks, infants’ looking behaviour was recorded using a Tobii 1750 eye-tracker (see the electronic supplementary material). In the two standardized assessments and the PCI, recoding was via video camera and microphone, onto digital videotape.

In the *face scanning task* [19], infants were presented with videos of female faces displaying four different dynamic sequences, each lasting approximately 16 s: (i) the eyes displayed gaze shifts, (ii) the mouth displayed vowel articulation movements, (iii) the hands positioned near the face displayed upward to downward motion, (iv) the eyes, mouth and hands moved displaying a *peek-a-boo* sequence. Each of these was preceded by a 5 s baseline period where the face was still. Pseudorandom presentation continued for a maximum of eight total trials per infant (two per sequence). Areas of interest were defined around the eye and mouth region. Trials were excluded if less than 1 s of data was accumulated. An eye–mouth index (EMI) was calculated as (looking time to the eyes − looking time to the mouth)/total looking time to the eyes and mouth. EMIs were then averaged for the static baseline period and for the dynamic period. Each of four dynamic sequences was analysed separately in a follow-up analysis (see the electronic supplementary material).

In the *gaze-following task* [3,18], infants observed a female actor seated in front of a table with two objects on top of it; one to the left and one to the right. The actor then looked at one of the objects, with the direction of gaze counterbalanced across trials. Each infant viewed 12 trials. The differential looking score (DLS), which is commonly used to assess gaze-following behaviour [3,20,21], was then calculated as the difference between the number of trials in which infants first looked at the object being looked at by the actor (i.e. the congruent object) and the trials in which infants looked at the other (i.e. incongruent) object. The number of incongruent trials was subtracted from the number of congruent trials, which was then divided by the sum of two types of trial to derive the DLS. To measure infants’ attention to the object looked at by the actor, looking time on the congruent object in those trials in which infant followed the gaze (i.e. looked at congruent object first) were averaged for each infant to calculate the gaze time.

The *Autism Observation Scale for Infants* (*AOSI*) [22] involves a semi-structured play assessment with an unfamiliar adult, originally designed to assess early behavioural manifestation of ASD in infants with a family history of autism. This was administered because of reports of increased prevalence of autistic-like behaviours in blind children [7], and in children who have experienced severe environmental adversity in their early development [6].

The *Mullen Scales of Early Learning* (*MSEL*) [23] is a standardized, direct assessment of verbal and non-verbal abilities for children from birth to 6 years of age. It was used to assess the general developmental level of infants at each visit. Scores across four domains—Visual Reception, Fine Motor, Receptive Language and Expressive Language—are combined to yield an overall Early Learning Composite (ELC; *M* = 100, s.d. = 15). Gross motor skills are also assessed but do not contribute to the ELC.

Short periods of naturalistic *PCI* were video-recorded in the laboratory. Dyads were given a box containing a small number of age-appropriate toys, and parents were asked to play as they normally would at home, using the toys if desired. Infant communication behaviours were later coded across a 6 min sample of the interaction, beginning when the researchers left the testing room and the parent and the infant were alone to play. Each infant communication act was identified and coded based on the social-communication protocol of Clifford *et al*. [24]. Codes were assigned for the communicative forms used in conveying the act (e.g. eye contact, vocalization, gesture, etc.). The following communication forms were retained for analysis: vocalization (i.e. non-verbal vocalization, approximations and single words), action (i.e. communicative movement of an object or of the infant's own body) and face gaze (i.e. eye contact or three-point gaze switch between an object and the parent's face). Other forms were coded (e.g. pointing, other gestures, following the parent's gaze/point and giving/showing an object to the parent) but not included in the analyses due to infrequent occurrence even in the large group of control infants. Coding of all footage was undertaken by an independent rater, blind to all information about participants (including group membership, age at visit and other data collected) and to the study aims/hypotheses. One of the authors (K.H.) coded footage for a random sample of control infants (*n* = 17 clips) as well as all the SIBP footage (*n* = 10 clips), to assess inter-rater reliability. Intra-class correlation coefficients (ICCs) across each of the retained form codes were very high (vocalization ICC = 0.95, action ICC = 0.87 and face gaze ICC = 0.87).

A follow-up home visit with the SIBP group, when aged between 24 and 47 months, included the *MSEL, Vineland Adaptive Behaviour Scale* (*VABS*)*, Autism Diagnostic Observation Scale-Generic* (*ADOS-G*)*, Autism Diagnostic Interview-Revised* (*ADI-R*) *and Social and Communication Questionnaire* (*SCQ*). The VABS [25] is a parental survey designed to assess everyday adaptive behaviour. The ADOS-G [26], ADI-R [27] and SCQ [28] are designed to assess symptoms of ASD. The ADOS-G is a structured behavioural observation, while the ADI-R is a structured parental interview and SCQ is a parental questionnaire. Like the AOSI undertaken at the infant laboratory visits, the ADOS-G also serves to provide an assessment of social interaction and communication skills with a sighted adult.

## 3. Results

### (a) Face scanning

**Figure 1.**
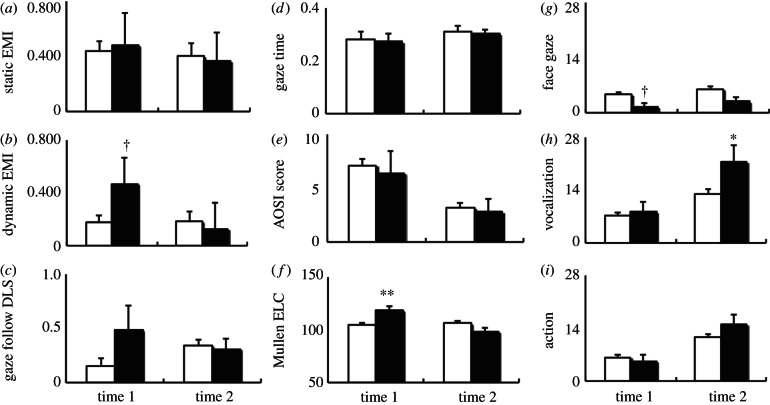
Results of sighted infants of blind parents (SIBP, filled bars) and control infants (control, unfilled bars) in (*a,b*) the face scanning task, eye–mouth index (EMI) in (*a*) static and in (*b*) dynamic conditions, in (*c,d*) the gaze-following task, (*c*) differential looking score (DLS) and (*d*) gaze time, (*e*) AOSI total score, (*f*) Mullen early learning composite score (ELC) and in (*g–i*) the parent–child interaction, the frequency of (*g*) face gaze, (*h*) vocalization and (*i*) action at time 1 (6–10 months) and time 2 (12–15 months). **; *p* < 0.01, *; *p* < 0.05, †; *p* < 0.1, Error bars: s.e.

At time 1 (6–10 months old), EMIs did not differ between groups when viewing static faces (*t*_50_ = 0.198, *p* = 0.844, *d* = 0.09; figure 1*a*), even though one SIBP infant scored slightly below 1.5 s.d. of the control infants’ mean score (table 1). EMIs were somewhat higher in SIBP (indicating more looking to the eyes than to the mouth) when viewing dynamic faces (*t*_50_ = 1.692, *p* = 0.097, *d* = 0.81; figure 1*b*) compared with controls. This non-significant trend was due to two SIBP infants scoring more than 1.5 s.d. above the control infants’ mean score (table 1). No infants scored below 1.5 s.d. of the control group mean when viewing dynamic stimuli. Both groups performed similarly at time 2, with all SIBP infants scoring within 1.5 s.d. of the control infant mean (all *t* < 0.25, all *p* > 0.80, all *d* < 0.13).

**Table 1.** Individual scores of SIBPs on various measures.

	time	control			female			male	
		*N*	mean	s.d.	SIBP01	SIBP02	SIBP03	SIBP04	SIBP05
face scanning									
EMI static	1	47	0.437	0.497	−0.332^b^	0.980	0.392	0.838	0.537
	2	41	0.405	0.564	0.493	0.634	−0.076	0.909	−0.108
EMI dynamic	1	47	0.176	0.362	−0.150	0.960^a^	0.232	0.792^a^	0.516
	2	41	0.186	0.473	0.105	0.214	−0.479	0.745	0.061
gaze following									
DLS	1	38	0.151	0.459	1.000^a^	1.000^a^	0.333	0.273	−0.167
	2	38	0.344	0.343	0.167	0.429	0.500	0	0.455
fixation duration	1	37	0.283	0.172	0.325	0.228	0.259	0.194	0.362
	2	37	0.310	0.145	0.292	0.293	0.319	0.349	0.262
social skills									
AOSI total score	1	50	7.12	4.074	6	8	1^b^	13	4
	2	48	3.17	3.251	5	4	0	5	0
Mullen scales of early learning									
early learning	1	50	104.42	11.31	122^a^	123^a^	125^a^	106	117
composite score	2	47	106.11	15.726	97	89	107	94	105
parent–child interaction									
face gaze	1	45	4.69	4.136	1	0	5	0	2
	2	46	6.00	5.198	7	2	3	0	2
vocalization	1	43	7.24	5.328	9	3	2	10	17
	2	45	13.02	8.142	24	15	27^a^	9	33^a^
action	1	45	6.30	3.834	12	4	2	5	3
	2	46	11.61	5.965	17	10	20	8	21
follow-up									
age assessed (months)					47	41	45	31	24
Mullen ELC score					113	130	117	74	116
VABC adaptive behaviour scale					117	111	108	97	107
ADOS-G communication subscale					0	2	2	0	1
ADOS-G social subscale					0	0	1	0	3
ADOS-G diagnosis					no^c^	no^c^	no^c^	no^c^	no^c^
ADI-R diagnosis					no^c^	no^c^	no^c^	no^c^	no^c^
SCQ total score					4	0	0	3	0

^a^scores above 1.5 s.d. of the mean of the control infants.

^b^scores below 1.5 s.d. of the mean of the control infants.

^c^not ASD [26,29].

### (b) Gaze following

As a group, SIBP followed the actor's gaze as frequently as, and then fixated on the gazed object as long as did control infants, across both visits (all *t* < 1.53, all *p* > 0.10, all *d* < 0.74; figure 1*c*). However, at time 1, two individual SIBP infants showed heightened DLS (indicating greater looking to the gazed object), above 1.5 s.d. of the mean of control infants (table 1). All the SIBP infants scored within 1.5 s.d. of the mean of the control infants for Gaze Time at time 1, and both DLS and Gaze Time at time 2 (figure 1*d*).

### (c) Autism observation scale for infants

SIBP were not observed to show atypical social behaviour at either of the two visits, and their AOSI scores were within the range of control infants (all *t* < 0.38, all *p* > 0.70, all *d* < 0.33; figure 1*e*). All the SIBP scored within 1.5 s.d. of the mean of the control infants. No atypicality of eye contact was observed in interaction with administrators (i.e. all SIBP scored 0 on the ‘eye contact’ codes).

### (d) Mullen scales of early learning

At time 1, SIBP showed significantly higher ELC than control infants (*t*_53_ = 2.730, *p* = 0.009, *d* = 1.30), demonstrating a *more advanced* developmental level (figure 1*f*). Four of the five infants scored above the 85th percentile, with three scoring above the 90th percentile of the normative data (table 1). Follow-up analyses of subscales demonstrated that the group difference at time 1 was most prominent in the visual reception subscale, which reached significance (*t*_53_ = 6.37, *p* < 0.001, *d* = 1.65). All five SIBP scored above the 85th percentile, with four scoring above the 95th percentile of the normative data. At time 2, ELCs did not differ between groups (*t*_50_ = 1.075, *p* = 0.287, *d* = 0.52), with all the SIBP infants scoring within 1.5 s.d. of the control group mean score, and scoring between the 23rd and 68th percentiles of the normative data.

### (e) Parent–child interaction

At time 1, SIBP showed marginally fewer instances of face gaze towards their blind parent than did control infants towards their sighted parent (*t*_48_ = 1.678, *p* = 0.10, *d* = 0.81; figure 1*g*). At an individual level, all SIBP infants scored within 1.5 s.d. of the mean of the control infants. At time 2, SIBP used significantly more frequent vocalization than did control infants (*t*_48_ = 2.208, *p* = 0.032, *d* = 1.06; figure 1*h*). Two SIBP infants scored above 1.5 s.d. of the mean of controls (table 1). No other group differences on infant communicative form approached significance within the PCI samples (all *t* < 1.27, all *p* > 0.21, all *d* < 0.60; figure 1*g–i*) with all SIBP scoring within 1.5 s.d. of the mean of the control infants across these.

### (f) Follow-up assessments

SIBP assessment scores at the follow-up visit were compared with the standardized/normative data available for each measure (table 1). Individuals’ scores on the ADI-R and SCQ were all well below the instrument cut-offs for ASD, as were the ADOS-G total algorithm scores and the domain subscores for communication and reciprocal social interaction symptoms. No atypicality of eye contact was observed in interaction with administrators (i.e. all SIBP scored 0 on the ‘eye contact’ codes). All of the SIBP scored within the average range on the VABS (i.e. between the 42nd and 87th percentile). Four out of 5 SIBP scored well above average on the MSEL (i.e. ELC above the 80th percentile), while the remaining SIBP child scored well below average on the MSEL (4th percentile).

## 4. Discussion

Our study provides the first empirical, systematic and longitudinal investigation of infants reared with specific reduced experience of eye contact and gaze behaviour owing to blindness in the primary carer. The results clearly demonstrated that no SIBP showed any autistic-like behaviours during the early infant and toddler years of life, indicating that early and ongoing interaction with a blind primary carer is not associated with clear and pervasive/persistent atypicalities in social-communication skills development. Results from our experimental tasks also failed to show any overall decrease or weakened skills in the specific use of eye contact or ability for gaze following compared with the control infants. These results are consistent with existing observational studies, but demonstrate more conclusively that SIBP show largely typical social-communication skills development.

Interestingly, we found that SIBP infants did, however, show a tendency to direct their own eye gaze differently towards their blind mothers when compared with sighted strangers. Specifically, the analyses of PCI demonstrated that SIBP tended to direct fewer gazes towards the face of their blind parents than did controls towards their sighted parents at time 1, and they used more vocal communication than did controls at time 2. In contrast, analyses of eye-tracking studies while the SIBP infants watched other unfamiliar adults showed no such reduction of attention to the eyes and face. Some SIBP even showed *greater* eye fixation or gaze following than controls, during the latter half of the first year of life. Those infants who showed greater eye fixation did not fully overlap with those who showed greater gaze following, suggesting that this tendency is not just driven by one or two ‘exceptional’ infants. Furthermore, no atypicality of eye contact was observed in interaction with administrators of the AOSI or ADOS-G assessments. We also observed a typical overall level of PCI from all the participants. Further studies should quantitatively assess the different modes of communication used, and their relation to the development of infants’ social-communication skills.

The profile of overall general development that we observed (as assessed by the MSEL) was somewhat unexpected. All the SIBP scored above the 85th percentile at time 1, driven mainly by high scores in visual reception (see the electronic supplementary material), which assesses visual memory and attention in this age range. The ELC scores then moved to fall to within the average range at time 2. At follow-up, when SIBP individuals were aged between 24 and 47 months, four out of the five scored above the 80th percentile.

Taken together, these results suggest that being reared with reduced experience of eye contact and gaze behaviour from the primary carer does not preclude sighted infants from developing typical gaze processing and social-communication skills. Perhaps the most striking feature of our data is that there was a tendency for the general developmental abilities, mainly in the areas of visual memory and attention, to be advanced in SIBP infants around the second half of the first year of life. They performed typically (or in the same way as sighted infants of sighted parents) when observing or interacting with sighted adults, but changed their behaviour adaptively when interacting with their own blind parents. Several studies have shown that the need to switch between spoken languages enhances various aspects of the development of infants growing up bilingually, or serves as a protective factor in the face of deprivation [30]. Our finding of higher developmental scores in the SIBP group in the latter half of the first year of life (as measured by the Mullen ELC) is consistent with the cognitive gains observed in bilingually exposed infants around the same age range [31]. In other words, the necessity to switch between visual and auditory channels of social communication when interacting with different adult partners may result in the facilitation of other aspects of development in SIBP. Interestingly, the gains in visual reception scores are present in time 1 and in the follow-up, but not in time 2. Further studies will be necessary to understand whether this reflects the fundamental course of cognitive development in SIBP, or whether it depends on the specific items used to test children at different ages.

The current results provide unique insights into the effects of postnatal environment on the development of infant social-communication skills. Firstly, the results indicate that infants can learn to change their sensory channels for social communication to adapt to their blind parents, suggesting some degree of plasticity in the development of non-verbal communication. Secondly, the results demonstrate that reduced visual communication experiences, such as contingent response to eye contact or gaze following, does not necessarily diminish an infant's capacity for gaze processing. This overall pattern of data appears to be only partially consistent with the nativist perspective, as this predicts no plasticity in response to the variability in postnatal environment. Some aspects of the data, such as the development of PCI, are consistent with the affective learning perspective as this predicts progressive decline of the interest in non-rewarding eye contact from the mother. However, this viewpoint does not explain their typical eye contact with sighted adults. Thus, the overall pattern of results may be best explained by the interactive specialization account, as this predicts that infants will develop different forms of specialization, depending on their individual experience and unique input from their blind parents [10]. However, we do not exclude the possibility that other mechanisms could contribute to parts of developmental profile we observed, such as the development of gaze-following behaviour [14].

Conclusions from the current study must be limited by the size of our SIBP sample. Nevertheless, some of the observed effects were large enough to reach statistical significance. Another limitation is the difficulty in controlling for heterogeneity within the SIBP group, such as the degree of sight in the father and the overall level of experience with other sighted adults, as well as background factors known to influence child development. Future studies with larger samples will be essential to better understand the role of interaction with sighted and blind adults for the development of non-verbal communication, gaze processing, social skills and other broader cognitive domains. In particular, it is essential to establish the role of the interaction with sighted adults and/or older siblings on the development of SIBP. In addition, further investigation of any subtle differences in the way SIBP communicate with sighted adults is merited. Further studies would also be beneficial into the way in which developmental changes in the infant brain are related to differential experience of adult gaze, and the subsequent effects on development of non-verbal communication skill.
